# Matching accuracy between CT images and intraoral surface scans using glass-ceramic markers compared to gutta-percha markers

**DOI:** 10.1186/s40729-026-00672-8

**Published:** 2026-02-23

**Authors:** Yoshiyuki Nakano, Takuya Mino, Yoko Kurosaki, Hiroaki Shimizu, Mariko Nishizaki, Kenji Maekawa

**Affiliations:** 1 Department of Removable Prosthodontics and Occlusion, School of Dentistry, Osaka Dental University, 1-5-17 Otemae, Chuo-ku, Osaka, 540-0008 Japan; 2 Department of Oral Rehabilitation and Implantology, Okayama University Hospital, 2-5-1 Shikata-cho, Okayama, 700-8525 Japan; 3 Shimizu Dental Clinic, 7-1-9 Kamisawa-dori, Hyogo-ku, Kobe, 652-0046 Japan

**Keywords:** Implant placement, Accuracy, Surgical guide, Fiducial markers, Artifact, Glass ceramics, Gutta-percha

## Abstract

**Purpose:**

This study investigated the accuracy of matching computed tomography (CT) images and intraoral surface scans using glass-ceramic (GC) and conventional gutta-percha (GP) markers.

**Methods:**

A mandibular master model of the right posterior edentulous region (teeth 45–47) was prepared with three training implants, each with a scan body fixed at the anterior central, left posterior, and right posterior sites. The model was scanned 10 times using an intraoral scanner, and 10 CT matching templates with six GP markers (GPCTMTs) and 10 with six GC markers (GCCTMTs) were fabricated. Each template was mounted on the model for CT imaging and intraoral scanning under air conditions. CT imaging was also performed with the model immersed in water to simulate intraoral scattering. A dentist blinded to the study purpose used implant simulation software for matching, which was performed with GP with six markers (GPCTMT) and GC with three (GCCTMT-3) or six (GCCTMT-6) markers. The three-dimensional discrepancy (matching error) between the implant apex positions on CT images and corresponding positions from surface data was measured automatically, and the median of the three sites served as the representative value. Group comparisons were performed using the Steel-Dwass test.

**Results:**

Under both air and water conditions, GCCTMT-3 (air: 0.61, water: 0.65) and GCCTMT-6 (air: 0.58, water: 0.57) demonstrated significantly lower matching errors (mm) than those in GPCTMT (air: 1.98, water: 1.83).

**Conclusions:**

This model-based study suggests that GC markers provide greater matching accuracy than GP markers for CT-surface data integration.

## Background

The fabrication methods for computer-aided design/computer-aided manufacturing (CAD/CAM) static surgical guide plates (SGPs) for dental implant placement can be broadly categorized into three approaches: the single computed tomography (CT) scan method (SCT) [1, 2], the double CT scan method (DCT) [3, 4], and the modified single CT scan method (MSCT) [5]. The SCT method involves matching CT images (Digital Imaging and Communications in Medicine; DICOM data) with intraoral surface scans (stereolithography; STL data) based on crown morphology. The DCT method involves matching CT images (DICOM) acquired with the template in place with CT images (DICOM) of the template alone, using reference markers attached to the template. The MSCT method involves matching CT images (DICOM) acquired with the template in place with intraoral surface scans (STL) obtained with the template in place, using reference markers attached to the template.

We focused on glass-ceramic (GC) materials, which produce minimal artifacts during CT imaging [6], and developed a novel MSCT method in which CT images (DICOM) are matched with intraoral surface scans (STL) using GC markers [5]. In a retrospective study involving 183 cases and 485 implants, we reported that SGPs fabricated using the MSCT method allowed more accurate implant placement according to the preoperative simulation, compared to those fabricated using the DCT or SCT methods [7]. However, the improved accuracy of SGPs fabricated with the MSCT method remains unclear, whether due primarily to the image-matching process between CT image (DICOM) and intraoral surface scans (STL) using reference markers, or attributed to the intrinsic properties of the GC marker material itself.

In recent years, the MSCT method has been increasingly applied in clinical practice, particularly in cases involving multiple missing teeth or numerous prosthetic restorations, which can serve as sources of scattering and beam-hardening artifacts. Although previous reports have indicated that gutta-percha (GP) markers produce minimal artifacts when cone-beam CT (CBCT) imaging is performed in air [8], other studies have shown that artifacts can occur when GP markers are used intraorally in the presence of surrounding tissues and restored teeth [9, 10]. Thus, uncertainty remains regarding whether accurate matching between CT images (DICOM) and intraoral surface scans (STL) can be achieved using GP markers as reference points. Demonstrating that GC markers allow more precise matching between CT images and intraoral surface scans compared to GP markers would provide a more reliable foundation for fabricating SGPs, ultimately contributing to safer, higher-quality implant treatments.

Therefore, this study aimed to compare the matching accuracy of CT images and intraoral surface scans using GC versus GP reference markers. The null hypothesis stated that no difference would exist in matching accuracy between DICOM images and STL data when aligned with either type of reference marker.

## Methods

### Preparation of the master model and fabrication of computed tomography matching templates (CTMTs)

**Fig. 1:**
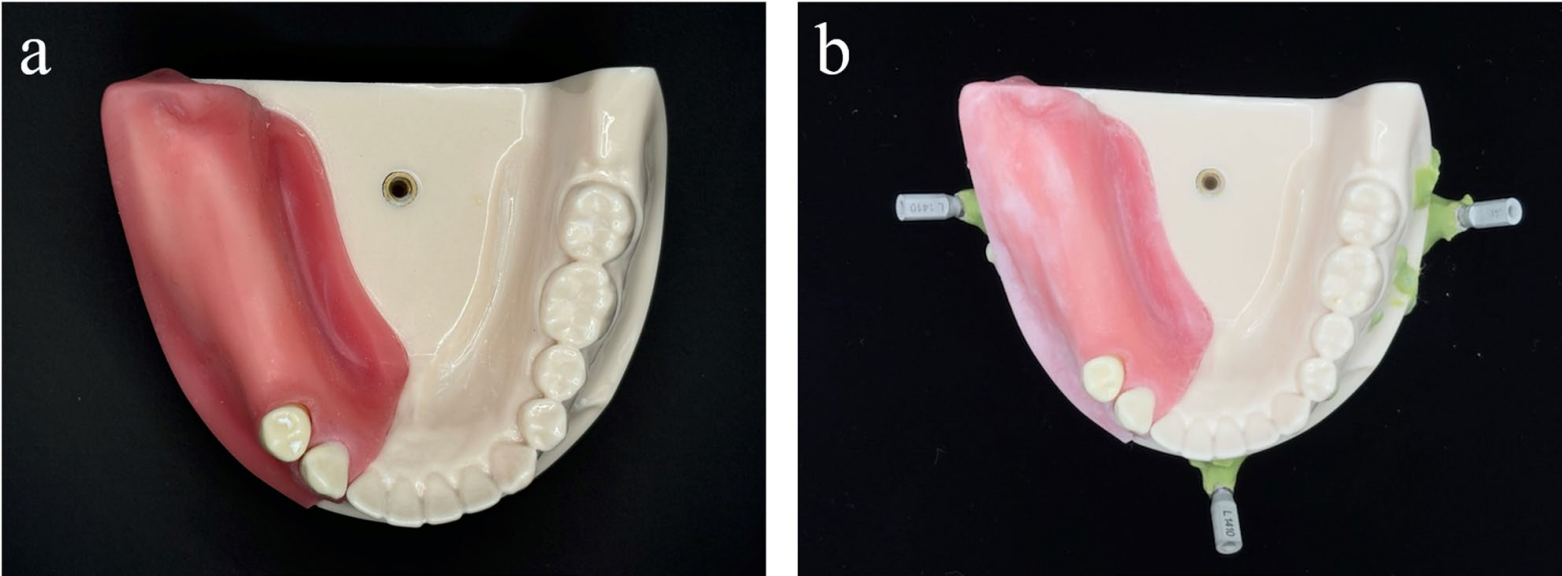
Preparation of the master model (simulated oral cavity). **a** Master model. **b **Master model with training implants connected to scan bodies

A commercial mandibular model with missing teeth in the right posterior region (P9FE-IMP.6; NISSIN Dental Products Inc., Kameoka, Japan) was used as the master model (Fig. 1a). To assess the accuracy of matching between CT images (DICOM) and simulated intraoral surface scans (STL), a dentist (TM), who was informed of the study objectives, placed three training implants (BL 4.1 × 10 mm; Straumann, Basel, Switzerland) at the left posterior, anterior central, and right posterior sites of the master model. The implants were fixed with resin (FIXPEED; GC Corporation, Tokyo, Japan), and scan bodies (Titanium Scan Body BL RC; Straumann, Basel, Switzerland) were connected to the training implants (Fig. 1b).

An intraoral scanner (Primescan; Dentsply Sirona, Charlotte, NC, USA) was used by TM to perform 10 scans of the master model, producing 10 simulated intraoral surface scan datasets (STL). These datasets were imported into computer-aided design (CAD) software (Geomagic Freeform Plus™, 3D Systems, Rock Hill, USA), where a CTMT was designed for each dataset. The designed CTMTs were fabricated using a 3D printer (ProJet MJP 2500; 3D Systems, Rock Hill, USA), with two resin CTMTs produced for each dataset.

**Fig. 2:**
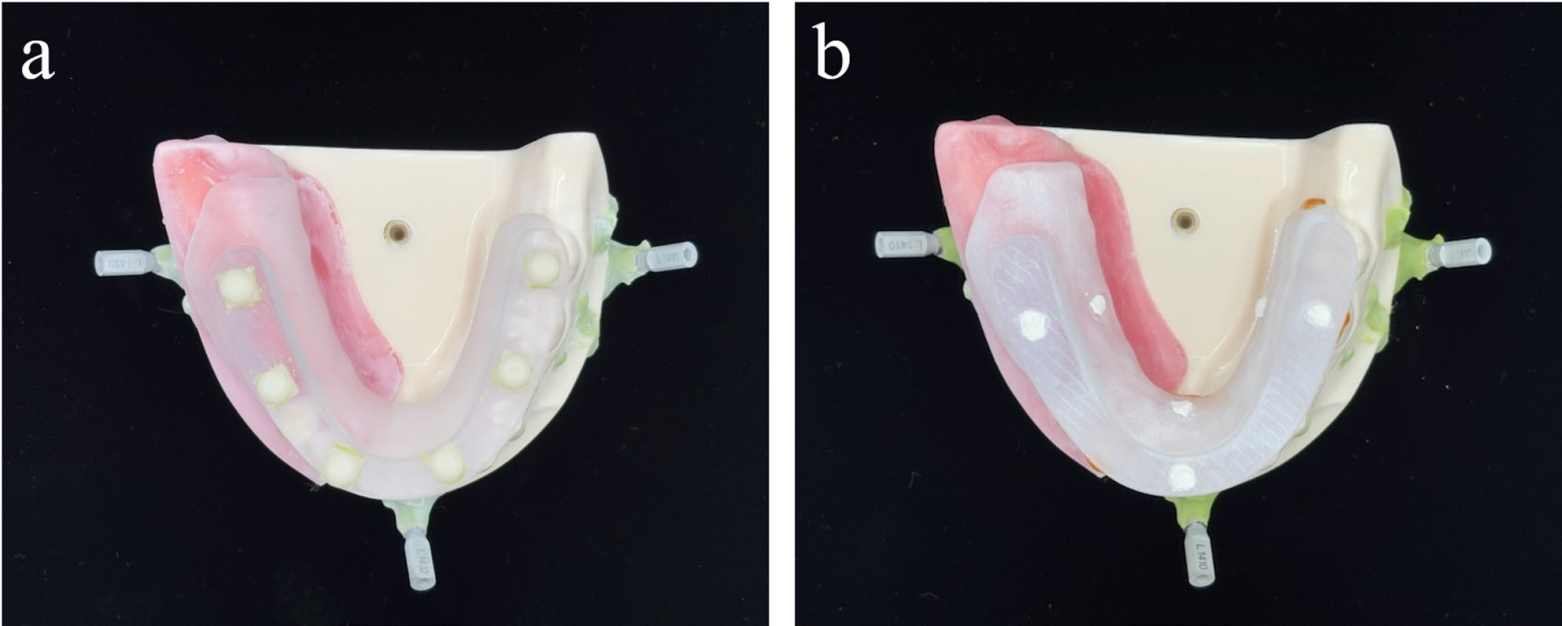
Fabricated CT matching templates (CTMTs). **a** GCCTMT mounted on the master model. **b** GPCTMT mounted on the master model

Of the two CTMTs fabricated per dataset, one was prepared with six GC markers (spherical, approximately 5 mm in diameter, with five 2-mm hemispherical concavities), and the material used was a GC primarily composed of lithium disilicate. However, due to patent restrictions, the trade names of the raw materials used for GC fabrication and detailed information about other components cannot be disclosed. These markers were attached to the occlusal surface (GCCTMT; Fig. 2a). The other CTMT was prepared using six GP markers (cylindrical, 5 mm in diameter, and 2 mm thick; TEMPORARY STOPPING, GC Corporation, Tokyo, Japan), with three markers attached to the occlusal surface and three to the lingual surface (GPCTMT; Fig. 2b). A total of 10 GCCTMTs and 10 GPCTMTs were fabricated, resulting in 20 CTMTs.

### Acquisition of CT images and simulated intraoral surface scans

**Fig. 3:**
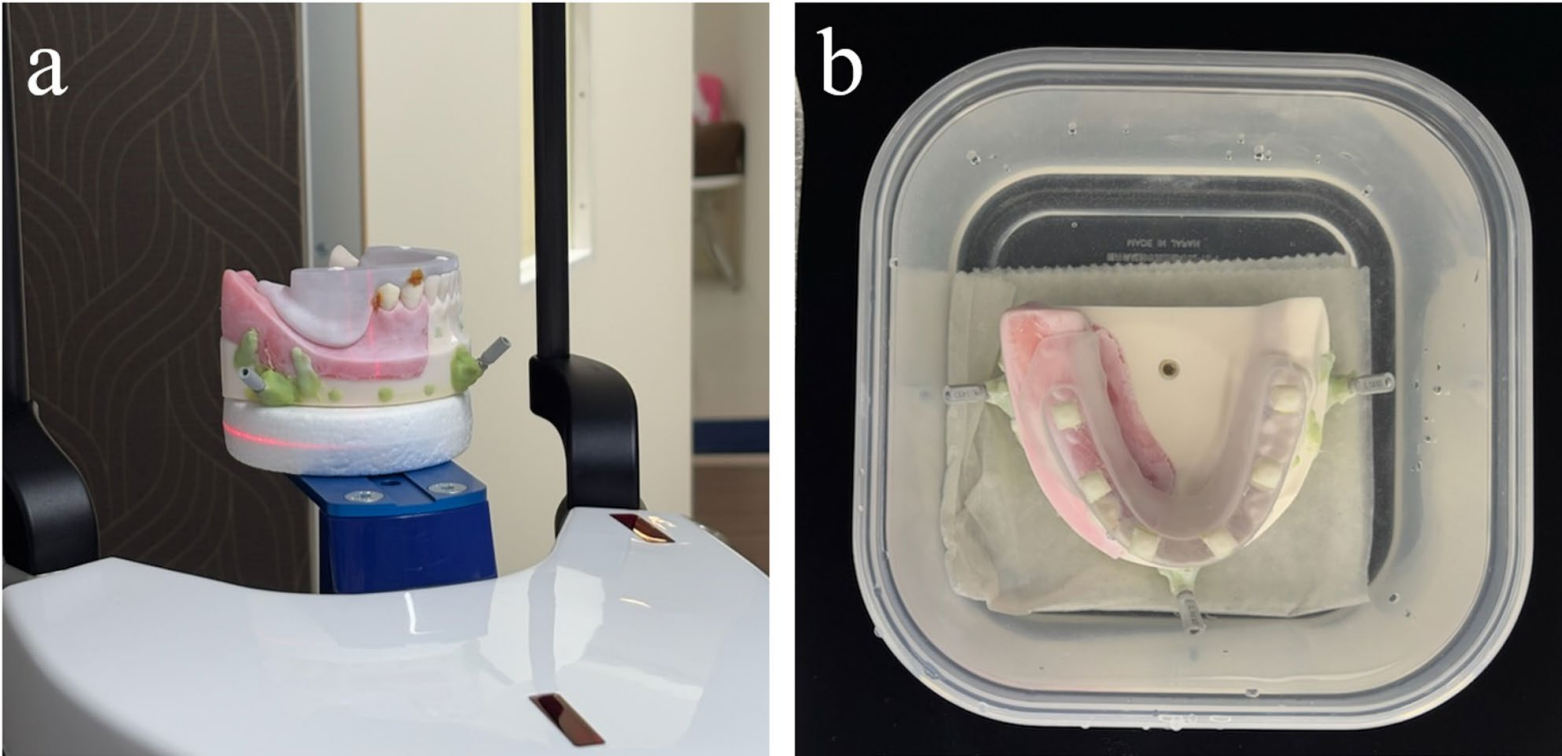
Cone-beam (CBCT) imaging conditions. **a** Under air conditions, the master model with CTMT was directly placed in the CBCT unit. **b** Under water conditions, the master model with CTMT was immersed in a container filled with water and placed in the CBCT unit

Each CTMT was sequentially fitted to the master model, followed by cone-beam CT (CBCT) scanning (ProMax 3D Plus; PLANMECA, Helsinki, Finland) and intraoral scanning (Trios3^®^; 3Shape, Copenhagen, Denmark). Simulated intraoral surface scan data (STL) and CT images (DICOM) were obtained. A single dentist experienced in intraoral scanning (TM) performed the optical scans, acquiring 10 datasets from both the GPCTMT and GCCTMT. CBCT imaging was performed by another dentist (HS) under two conditions: air (Fig. 3a) and water to simulate intraoral scattering (Fig. 3b) [11–13]. Consequently, 40 CT datasets were acquired (10 GPCTMT-air, 10 GPCTMT-water, 10 GCCTMT-air, and 10 GCCTMT-water). The CBCT imaging parameters were as follows: field of view (FOV) 200 × 100 mm, voxel size 0.2 mm (HD mode), tube voltage 90 kV, tube current 8 mA, and exposure time 18s. It should be noted that different intraoral scanners were used for the initial optical impression of the master model (Primescan) and for the experimental scans (Trios3). This decision was made solely for reasons of workflow convenience and equipment availability at each stage of the study. As the initial scan served exclusively for CAD design of the CTMTs and not included in the accuracy assessment, the use of different scanners does not influence the matching accuracy results.

### Matching of CT images and simulated intraoral surface scans

The acquired CT images (DICOM) were imported into the simulation software (coDiagnostiX^®^; Dental Wings Inc., Montreal, Canada). Threshold values were adjusted to ensure the clear visualization of the reference markers (GC or GP). Matching of the CT images (DICOM) and simulated intraoral surface scans (STL) was then performed using a standard procedure based on the reference markers. For each simulated intraoral surface scan (STL), matching was conducted for both air and water CT datasets.

**Fig. 4:**
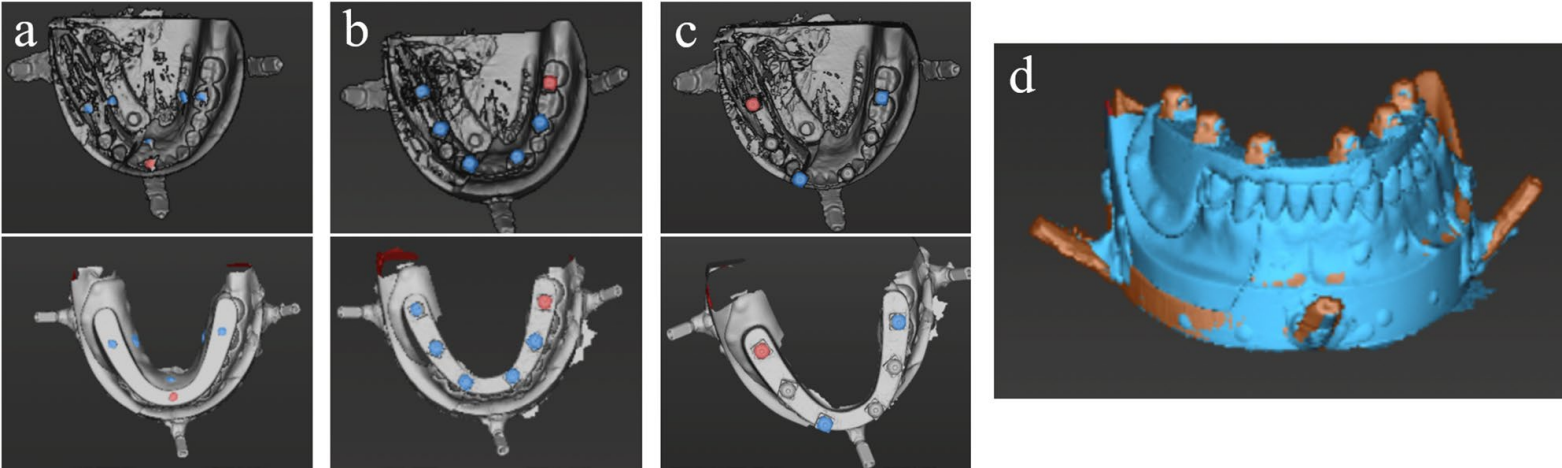
Matching between CT images and simulated intraoral surface scans using reference markers. **a** Using six GP markers as reference markers (upper: CT image; lower: simulated intraoral surface scan). **b** Using six GC markers as reference markers (upper: CT image; lower: simulated intraoral surface scan). **c** Using three GC markers as reference markers (upper: CT image; lower: simulated intraoral surface scan). **d** CT image and simulated intraoral surface scan after matching using six GC markers

For the 20 GPCTMT datasets, all six markers were used for matching (Fig. 4a). For the 20 GCCTMT datasets, matching was performed in two ways: using three markers (one anterior and one in each posterior region; GCCTMT-3) and using all six markers (GCCTMT-6) (Fig. 4b, c, d). A total of 60 matched datasets were obtained. Since the shape and position of the reference markers were identifiable by the software, blinding regarding the type of CTMT was not feasible. Therefore, one dentist (MN), who was blinded to the study objectives, performed all matching procedures.

## Measurement of matching accuracy between CT images and simulated intraoral surface scans

**Fig. 5:**
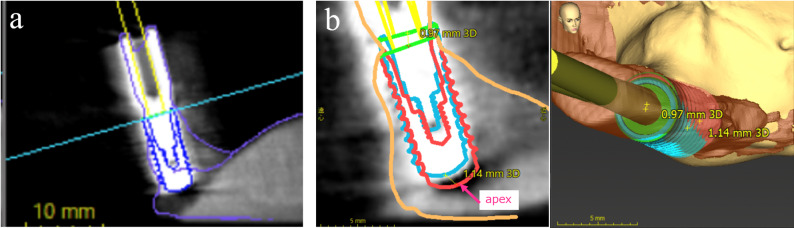
Measurement of matching error between CT images and simulated intraoral surface scans. **a** Placement of a virtual implant body onto the implant shadow on the CT image. **b** Measurement of matching error using the treatment evaluation function of the simulation software (blue: virtual implant on the CT image; red: implant reconstructed from the 3D surface-scan data of the scan body)

In the simulation software, a virtual implant was manually positioned to align with the radiographic image of the training implant in the CT data (DICOM) (Fig. 5a). The implant position was then automatically calculated based on the scan bodies of the simulated intraoral surface scan (STL) data. Using the software’s treatment evaluation function, the three-dimensional distance (matching error, in millimeters) between the apex of the training implant and that of the virtual implant was automatically measured (Fig. 5b). All measurements were performed by a single examiner (MN), who was blinded to the study objectives. To assess inter-examiner reliability, a second examiner, a board-certified implantologist (TM), independently measured 10 randomly selected cases from all datasets (*n* = 30).

### Statistical analysis

The inter-examiner reliability of the measurements was assessed using the intraclass correlation coefficient (ICC), based on 30 values obtained from 10 datasets measured by examiners MN and TM. To evaluate the matching accuracy between the CT images (DICOM) and simulated intraoral surface scans (STL), the three-dimensional deviations (mm) of the GPCTMT, GCCTMT-3, and GCCTMT-6 groups were calculated. These values were then compared between the imaging environments using the Steel-Dwass test. For each group, three-dimensional deviations (mm) were assessed for the left posterior, anterior central, and right posterior sites of the master model, as well as for the representative value, which was defined as the median of the three sites. ICC analyses were performed using SPSS for Windows (version 25; IBM, Japan), and the Steel-Dwass test was conducted using JMP (version 11; SAS, Japan). The significance level was set at *p* < 0.05.

The Steel–Dwass test was used to compare the three groups (GPCTMT, GCCTMT-3, and GCCTMT-6) within each imaging environment (air and water).

## Results

### Inter-examiner reliability of the measurement

The ICC for inter-examiner reliability was 0.985, indicating almost perfect agreement, according to the criteria of Landis and Koch [14].

### Matching accuracy of the GPCTMT, GCCTMT-3, and GCCTMT-6 groups under air conditions

Table 1 presents the matching accuracy for each group at the left posterior, anterior central, and right posterior sites, as well as the representative value (median of the three sites), along with the results of intergroup comparisons. No statistically significant differences in matching accuracy were observed between the groups at the left and right posterior sites. In contrast, at the anterior central site and for the representative value (median), the matching error in the GPCTMT group was significantly larger than that in the GCCTMT-3 and GCCTMT-6 groups.

**Table 1:** Matching accuracy of the GPCTMT, GCCTMT-3, and GCCTMT-6 groups under air conditions

		*n*	Median (Q1-Q3), mm	95%CI for median, mm	*p* value	
*Representative value*						
a	GPCTMT	10	1.98 (0.88–2.33)	0.96–2.40	a vs. b	**0.037**
b	GCCTMT-6	10	0.58 (0.49–0.69)	0.50–0.68	a vs. c	**0.037**
c	GCCTMT-3	10	0.61 (0.52–0.84)	0.49–0.82	b vs. c	0.752
*Left posterior site*						
a	GPCTMT	10	1.39 (0.40–2.32)	0.40–2.24	a vs. b	0.47
b	GCCTMT-6	10	0.71 (0.41–1.01)	0.44–0.96	a vs. c	0.880
c	GCCTMT-3	10	0.57 (0.49–1.02)	0.49–0.79	b vs. c	1.000
*Anterior central site*						
a	GPCTMT	10	1.82 (0.96–2.30)	1.01–2.31	a vs. b	**0.005**
b	GCCTMT-6	10	0.46 (0.37–0.57)	0.29–0.56	a vs. c	**0.013**
c	GCCTMT-3	10	0.62 (0.36–0.87)	0.39–0.91	b vs. c	0.492
*Right posterior site*						
a	GPCTMT	10	2.38 (0.94–3.12)	1.10–3.09	a vs. b	0.072
b	GCCTMT-6	10	0.64 (0.50–0.78)	0.50–0.79	a vs. c	0.066
c	GCCTMT-3	10	0.69 (0.52–1.50)	0.56–1.43	b vs. c	0.875

GP, gutta-percha; GC, glass-ceramic; CTMT, computed tomography matching template;GPCTMT, CTMT with six GP markers (all six markers used for matching);GCCTMT-6, CTMT with six GC markers (all six markers used for matching);GCCTMT-3, CTMT with six GC markers (three markers used for matching);Q1, first quartile (25th percentile); Q3, third quartile (75th percentile); CI, confidence interval

*p* values ≤ 0.05 are shown in bold

### Matching accuracy of the GPCTMT, GCCTMT-3, and GCCTMT-6 groups under water conditions

Table 2 presents the matching accuracy for each group at the left posterior, anterior central, and right posterior sites, as well as the representative value (median of the three sites), along with the results of intergroup comparisons. No statistically significant differences in matching accuracy were observed between the groups at the left posterior site. In contrast, at the anterior central and right posterior sites, as well as for the representative value (median), the matching error in the GPCTMT group was significantly larger than that in the GCCTMT-3 and GCCTMT-6 groups.

**Table 2:** Matching accuracy of the GPCTMT, GCCTMT-3, and GCCTMT-6 groups under water conditions

		*n*	Median (Q1-Q3), mm	95%CI for median, mm	*p* value	
*Representative value*						
a	GPCTMT	10	1.83 (0.82–2.01)	0.71–2.01	a vs. b	**0.025**
b	GCCTMT-6	10	0.57 (0.44–0.59)	0.45–0.59	a vs. c	**0.045**
c	GCCTMT-3	10	0.65 (0.56–0.74)	0.57–0.74	b vs. c	0.140
*Left posterior site*						
a	GPCTMT	10	1.44 (1.05–1.93)	0.86–1.93	a vs. b	0.055
b	GCCTMT-6	10	0.61 (0.55–0.78)	0.55–0.77	a vs. c	0.066
c	GCCTMT-3	10	0.70 (0.61–0.87)	0.60–0.86	b vs. c	0.516
*Anterior central site*						
a	GPCTMT	10	1.38 (0.54–2.01)	0.56–2.01	a vs. b	**0.022**
b	GCCTMT-6	10	0.43 (0.38–0.58)	0.36–0.57	a vs. c	**0.045**
c	GCCTMT-3	10	0.49 (0.40–0.59)	0.40–0.58	b vs. c	0.817
*Right posterior site*						
a	GPCTMT	10	1.90 (0.85–2.47)	0.94–2.63	a vs. b	**0.025**
b	GCCTMT-6	10	0.56 (0.47–0.71)	0.47–0.69	a vs. c	**0.045**
c	GCCTMT-3	10	0.69 (0.64–0.77)	0.62–0.77	b vs. c	0.140

GP, gutta-percha; GC, glass-ceramic; CTMT, computed tomography matching template;GPCTMT, CTMT with six GP markers (all six markers used for matching);GCCTMT-6, CTMT with six GC markers (all six markers used for matching);GCCTMT-3, CTMT with six GC markers (three markers used for matching);Q1, first quartile (25th percentile); Q3, third quartile (75th percentile); CI, confidence interval

*p* values ≤ 0.05 are shown in bold

## Discussion

The purpose of this study was to evaluate, at the model level, whether GP or GC provides higher matching accuracy as a reference marker material in the MSCT method, where the critical step involves matching between CT images (DICOM) and intraoral surface scans (STL). Since no previous studies have directly compared the matching accuracy of CT images (DICOM) and intraoral surface scans (STL) using GP and GC markers, the present study offers novel insights into this comparison.

### Measurement of matching accuracy between CT images and simulated intraoral surface scans

A distinctive feature of the evaluation method used in this study was the incorporation of training implants connected to scan bodies as reference standards, alongside the treatment evaluation function of the simulation software, which automatically measures three-dimensional discrepancies. Conventional methods for assessing the matching accuracy between CT images (DICOM) and intraoral surface scans (STL) generally rely on manual measurement of the three-dimensional distances between landmarks identified on the CT images of the model and the corresponding landmarks on the surface scans using the distance measurement tool in the simulation software [15]. In contrast, the method employed in this study required manual input only for positioning virtual implants onto the implant shadows on the CT images. All other steps were automated, utilizing digital evaluation techniques. This methodological approach contributed to nearly perfect inter-examiner reliability.

### Matching accuracy of the GPCTMT, GCCTMT-3, and GCCTMT-6 groups

Under all conditions, the matching errors for the GCCTMT-3 and GCCTMT-6 groups were generally smaller than those for the GPCTMT group, with statistically significant differences observed, particularly in the representative values under air (representative value and anterior central site) and water conditions (representative value, anterior central site, and right posterior site). These results indicate that the use of GC markers provides higher accuracy in matching CT images (DICOM) with intraoral surface scans (STL) compared to GP markers.

The first possible explanation for this finding is the difference in artifact formation between the two marker materials during CBCT imaging. Only a few studies have reported the number of artifacts generated by both materials within the same experimental framework. Kuo et al. reported that when evaluated in air, both GP and GC blocks produced extremely minimal and comparable artifacts [8]. However, as previous studies have indicated, artifact generation during CBCT imaging can be influenced by interactions with the surrounding environment [16, 17]. It is therefore plausible that GP markers are more susceptible to environmental influences than GC markers. Indeed, artifact generation from GP in root canals during CBCT imaging has been reported in several studies [9, 10], and this is also a common observation experienced by clinicians in daily practice. In contrast, the extent to which environmental factors affect artifact generation from GCs remains unclear, and further studies are warranted to clarify this issue.

A second possible explanation for the observed differences in matching accuracy is the variation in marker geometry. In this study, GC markers were designed with a more complex geometry compared to GP markers, which may have contributed to the higher accuracy observed in matching between CT images (DICOM) and intraoral surface scans (STL). However, due to the low strength and stiffness of GP [18, 19], it is difficult to maintain a complex geometry. This limitation should therefore be regarded as an inherent material property of GP markers. Although differences in matching accuracy were observed between GC and GP markers, the present study could not determine whether marker size contributed to these results. The GC and GP markers differed not only in volume but also in material properties and geometric configuration, all of which may influence marker detectability and artifact behavior on CBCT images. As it was not possible for these factors to be isolated within the current experimental design, the effect of marker size on matching accuracy remains inconclusive. Future studies that systematically vary marker dimensions while controlling for material characteristics will be necessary to clarify this issue. Although the GCCTMT-6 group showed a tendency toward smaller matching errors than the GCCTMT-3 group, the difference between the two was not statistically significant. Conceptually, an increased number of matching points is expected to improve matching accuracy. However, the high accuracy achieved by the GCCTMT-3 group may be attributed to the balanced placement of matching points in the anterior and bilateral posterior regions.

In this study, in addition to the air condition, a water condition was established, which is known to generate more scattered radiation [11–13]. Previous phantom-based studies have reported that increasing the water volume within the CBCT field of view enhances the scattering effect, thereby increasing the number of artifacts produced by the materials [20]. However, in the present study, no differences were observed in the trends of matching accuracy between CT images (DICOM) and intraoral surface scans (STL) under the two conditions. One possible explanation is that both GP and GC markers inherently produce minimal artifacts, making them less susceptible to the artifact-enhancing effects of scattered radiation. Furthermore, under the current experimental conditions, no high-CT-value materials, such as metals or zirconia, which are known to generate strong artifacts, were included within the CBCT field of view. Consequently, artifacts originating from non-marker sources were not amplified by scattered radiation and did not interfere visually with the matching process. Future studies should evaluate similar conditions using models restored with crowns fabricated from high-CT-value materials, such as metals or zirconia.

### Limitations

This study was conducted at the model level, and its findings should therefore be interpreted with caution. The clinical situation differs from the experimental setting, where various hard and soft tissues are present within the CBCT field of view. Furthermore, because the shapes of the GP and GC markers differed, visual blinding between the two marker types during the matching process was not feasible. However, to minimize the risk of bias, the matching process was performed by an examiner who was completely unaware of the study’s objectives. Moreover, as mentioned previously, differences in shape between the GP and GC markers cannot be excluded as a potential factor influencing the matching accuracy. However, the superior shape reproducibility and stability of the GC marker should also be considered an advantage. Additionally, the difference in marker geometry-spherical GC markers versus cylindrical GP markers-may also have affected marker detectability and contributed to the differences in matching accuracy observed in this study.

## Conclusions

Within the limitations of this model-based study, the use of GC markers was demonstrated to provide greater matching accuracy between CT images (DICOM) and intraoral surface scans (STL) than GP markers. This finding suggests that GC reference markers can improve the reliability of CT-surface data integration, thereby contributing to safer and more precise dental implant placement.

## Data Availability

The datasets generated and analyzed during the current study are available from the corresponding author on reasonable request.

## Abbreviations

CAD/CAM: Computer-aided design/computer-aided manufacturing
SGP: Surgical guide plate
CT: computed tomography
CB: Cone-beam
3D: Three-dimensional
SCT: Single computed tomography
DCT: Double computed tomography
MSCT: Modified single computed tomography
DICOM: Digital imaging and communications in medicine
STL: Stereolithography
CTMT: Computed tomography matching template
GP: Gutta-percha
GC: Glass-ceramic
ICC: Intraclass correlation coefficient
